# Supplementary material for: Nano3P-seq: transcriptome-wide analysis of gene expression and tail dynamics using end-capture nanopore cDNA sequencing
Source: Nat Methods. 2022 Dec 19;20(1):75–85. doi: 10.1038/s41592-022-01714-w (PMC9834059; doi:10.1038/s41592-022-01714-w)
Supplement: Supplementary file 1 — Supplementary Protocol, Supplementary Tables 1–3, and Supplementary Notes 1–2. [file 41592_2022_1714_MOESM1_ESM.pdf]

nature methods

Article https://doi.org/10.1038/s41592-022-01714-w

Nano3P-seq: transcriptome-wide analysis of gene expression and tail dynamics using end-capture nanopore cDNA sequencing

In the format provided by the authors and unedited

SUPPLEMENTARY INFORMATION

Nano3P-seq: transcriptome-wide analysis of gene expression and tail dynamics using end-capture nanopore cDNA sequencing

Oguzhan Begik[1,2,3], Gregor Diensthuber[1], Huanle Liu[1], Anna Delgado-Tejedor[1,4], Cassandra Kontur[5], Adnan Muhammad Niazi[6], Eivind Valen[6,7], Antonio J. Giraldez[5], Jean-Denis Beaudoin[8], John S. Mattick[2,9] and Eva Maria Novoa[1,4*]

[1] Centre for Genomic Regulation (CRG), The Barcelona Institute of Science and Technology, Dr. Aiguader 88, Barcelona 08003, Spain

[2] Garvan Institute of Medical Research, 384 Victoria St., Darlinghurst, 2010, NSW, Australia

[3] Faculty of Medicine, University of New South Wales, Sydney NSW 2052, Australia

[4] Universitat Pompeu Fabra, Dr. Aiguader 88, Barcelona 08003, Spain

[5] Department of Genetics, Yale University, USA

[6] Computational Biology Unit, Department of Informatics, University of Bergen, 5008 Bergen, Norway

[7] Sars International Centre for Marine Molecular Biology, University of Bergen, 5008 Bergen, Norway

[8] Department of Genetics and Genome Sciences, Institute for Systems Genomics, University of Connecticut Health Center, Farmington, CT, USA

[9] School of Biotechnology and Biomolecular Sciences, University of New South Wales, Sydney NSW 2052, Australia

* Correspondence to: Eva Maria Novoa (eva.novoa@crg.eu)

SUPPLEMENTARY PROTOCOL

Library Preparation protocol for Nano3P-Seq

1. **Without Barcoding**

Materials and consumables required:

- Direct cDNA Sequencing Kit (ONT, SQK-DCS109)
- Flow Cell Priming Kit (ONT, EXP-FLP001)
- AMPure XP Reagent (Agencourt , A63881)
- Blunt/TA Ligase Master Mix (NEB, M0367)
- TGIRT™-III Enzyme (InGex)
- RNase Inhibitor, Murine (NEB, M0314L)
- RNase Cocktail Enzyme Mix (ThermoFisher, AM2286)
- Qubit dsDNA HS Assay Kit (ThermoFisher, Q32854)
- 10 mM dNTP solution (ThermoFisher, R0191)
- 0.1 M DTT solution (ThermoFisher , 707265ML)
- 1.5 mL Eppendorf DNA LoBind Tubes (Eppendorf, 0030108051)
- 0.2 ml thin-walled PCR tubes (Starlab, A1402-3700)
- 1 M Nuclease-free Tris-Cl pH 7.5 (ThermoFisher, 15567027)
- 5 M Nuclease-free NaCl (ThermoFisher, AM9760G)
- 1 M Nuclease-free MgCl2 (ThermoFisher, AM9530G)
- Nuclease-free water (ThermoFisher, AM9922)
- Freshly prepared 70% ethanol in nuclease-free water

Oligos required :

Oligo Name	Sequence
D_DNA (Standard desalting)	/5Phos/CTTCCGATCACTTGCCTGTCGCTCTATCTTCN
R_RNA (RNase-Free HPLC)	rGrArArGrArUrArGrArGrCrGrArCrArGrGrCrArArGrUrGrArUrCrGrGrArArG/3SpC3/
CompA_DNA (Standard desalting)	GAAGATAGAGCGACAGGCAAGTGATCGGAAGA

Preannealing of the oligos

We need pre-anneal R_RNA and D_DNA oligos in order to be able to initiate template switching.

Reagent	Initial Concentration	Volume	Final Conc
R_RNA	10 uM	1 uL	1 uM
D_DNA	10 uM	1 uL	1 uM
Tris-Cl pH 7.5	0.1 M	1 uL	0.01 M
NaCl	0.5 M	1 uL	0.05 M
RNase Inhibitor, Murine		0.5 uL	
dH2O		5 uL	
Total		10 uL	

- Heat the mixture for 94°C for 1 mins and ramp down to 25°C at 0.1°C/s (in thermal cycler).

Reverse Transcription

Component	Initial Conc	Volume	Final Conc/Amount
5X Reaction Buffer	2.25 M NaCl, 25 mM MgCl2, 100 mM Tris-HCl, pH 7.5	4 uL	450 mM NaCl, 5 mM MgCl2, 20 mM Tris-HCl, pH 7.5
DTT	0.1 M	1 uL	5 mM
Pre-annealed oligos	1 uM	2 uL	0.1 uM
RNA		Up to 10 uL	50-100 ng
TGIRT	10uM- 200 Unit/ul	1-2 uL	500 nM- 1000 nM
RNase Inhibitor, Murine		1 uL	
Total		19 uL	

- *Pre-incubate at room temperature for 30 minutes, then add* ***1 ul of 10 mM dNTPs***
- Incubate at 60°C for 1 hour
- Inactivate the enzyme by incubating at 75°C for 15 mins
- Move reaction to ice

RNase treatment

- Add 1.5 ul RNase Cocktail Enzyme Mix to each tube
- 37°C 10 minutes incubation
- Move reaction to ice

Cleanup using Ampure XP Beads

- Mix the samples with the appropriate volume of beads (17 ul, 0.8 X keeps everything above 150 nt, good for getting rid of adapters)
- Mix the beads by flicking
- Incubate 10 minutes at room temperature
- Spin down the tube and place it on the magnet
- Remove the supernatant
- Add 70% freshly prepared 200 ul ethanol to the tube
- Incubate for 30 seconds at room temperature
- Remove the ethanol completely by spinning down the tube, placing back it on magnet and removing the liquid
- Air-dry the pellet for maximum 1 minute, do not let it dry out completely!
- Resuspend the beads in 16 ul water
- Incubate 5-10 minutes in RT
- Place the beads on magnet
- Transfer the supernatant into a new tube.
- Quantify 1 μl of eluted sample using a Qubit fluorometer
- Move to the next step

Annealing of Complementary DNA to VNP Oligo

This step is essential to have a double-stranded DNA oligo with an A overhang, which will initiate the ligation to the adapter

Components	Initial Concentration	Final Conc.	Volume
cDNA			15 uL
Tris-Cl pH 7.5	0.1 M	0.01 M	2.25 uL
NaCl	0.5 M	0.05 M	2.25 uL
CompA_DNA	10 uM	0.44 uM	1 uL
Water			2 uL
Total			22.5 uL

- Mix by flicking
- Heat the mixture for 90°C for 1 mins and ramp down to RT at 0.1°C/s (in thermal cycler).
- Mix the following

AMX Adapter Ligation

22.5 uL cDNA-complement mix
2.5 uL Adapter Mix (AMX adapter from the SQK-DCS109 kit)
25 uL Blunt/TA Ligase Mix

- Mix by flicking
- Spin down
- Incubate at RT for 10 minutes

Ampure XP Beads Cleanup

- Add 25 ul resuspended AMPure XP beads (0.5X) to the reaction and mix by flicking
- Incubate 10 minutes at room temperature
- Thaw Wash Buffer (WSB) and Elution Buffer (EB) at RT, mix by vortexing, spin down and place on ice. Check if the contents of each tube are clear of any precipitate.
- Spin down the tube and place it on the magnet
- Remove the supernatant
- Add 200 ul WSB to the beads. Close the tube lid, and resuspend the beads by flicking. Return the tube to the magnetic rack, allow beads to pellet and pipette off the supernatant
- Repeat the previous step.
- Remove the WSB completely by spinning down the tube, pipetting out the liquid and placing back it on magnet
- Air-dry the pellet for maximum 1 minute, do not let it dry out completely!
- Resuspend the beads in 13 ul Elution Buffer (EB)
- Incubate 5-10 minutes in RT
- Place the beads on magnet
- Place the elute in a 1.5 ml tube
- Quantify 1 μl of eluted sample using a Qubit fluorometer
- The prepared library is used for loading into the MinION flow cell. Store the library on ice until ready to load.
- Move to the “Prepare the library for loading” step.

2. **With Barcoding**

Materials and consumables required:

- Direct cDNA Sequencing Kit (ONT, SQK-DCS109)
- Native Barcoding Expansion 1-12 (EXP-NBD104)
- Flow Cell Priming Kit (ONT, EXP-FLP001)
- NEBNext Quick Ligation Module (E6056)
- AMPure XP Reagent (Agencourt , A63881)
- Blunt/TA Ligase Master Mix (NEB, M0367)
- TGIRT™-III Enzyme (InGex)
- RNase Inhibitor, Murine (NEB, M0314L)
- RNase Cocktail Enzyme Mix (ThermoFisher, AM2286)
- Qubit dsDNA HS Assay Kit (ThermoFisher, Q32854)
- 10 mM dNTP solution (ThermoFisher, R0191)
- 0.1 M DTT solution (ThermoFisher , 707265ML)
- 1.5 mL Eppendorf DNA LoBind Tubes (Eppendorf, 0030108051)
- 0.2 ml thin-walled PCR tubes (Starlab, A1402-3700)
- 1 M Nuclease-free Tris-Cl pH 7.5 (ThermoFisher, 15567027)
- 5 M Nuclease-free NaCl (ThermoFisher, AM9760G)
- 1 M Nuclease-free MgCl2 (ThermoFisher, AM9530G)
- Nuclease-free water (ThermoFisher, AM9922)
- Freshly prepared 70% ethanol in nuclease-free water

Oligos required :

Oligo Name	Sequence
D_DNA (Standard desalting)	/5Phos/CTTCCGATCACTTGCCTGTCGCTCTATCTTCN
R_RNA (RNase Free HPLC)	rGrArArGrArUrArGrArGrCrGrArCrArGrGrCrArArGrUrGrArUrCrGrGrArArG/3SpC3/
CompA_DNA (Standard desalting)	GAAGATAGAGCGACAGGCAAGTGATCGGAAGA

Preannealing of the oligos

We need pre-anneal R_RNA and D_DNA oligos in order to be able to initiate template switching.

Reagent	Initial Concentration	Volume	Final Conc
R_RNA	10 uM	1 uL	1 uM
D_DNA	10 uM	1 uL	1 uM
Tris-Cl pH 7.5	0.1 M	1 uL	0.01 M
NaCl	0.5 M	1 uL	0.05 M
RNase Inhibitor, Murine		0.5 uL	
dH2O		5 uL	
Total		10 uL	

- Heat the mixture for 94°C for 1 mins and ramp down to 25°C at 0.1°C/s (in thermal cycler).

Important note: Prepare one reaction for each sample/barcode

Reverse Transcription

Component	Initial Conc	Volume	Final Conc/Amount
5X Reaction Buffer	2.25 M NaCl, 25 mM MgCl2, 100 mM Tris-HCl, pH 7.5	4 uL	450 mM NaCl, 5 mM MgCl2, 20 mM Tris-HCl, pH 7.5
DTT	0.1 M	1 uL	5 mM
Pre-annealed oligos	1 uM	2 uL	0.1 uM
RNA		Up to 10 uL	50-100 ng
TGIRT	10uM- 200 Unit/ul	1-2 uL	500 nM- 1000 nM
RNase Inhibitor, Murine		1 uL	
Total		19 uL	

- *Pre-incubate at room temperature for 30 minutes, then add* ***1 ul of 10 mM dNTPs***
- Incubate at 60°C for 1 hour
- Inactivate the enzyme by incubating at 75°C for 15 mins
- Move reaction to ice

RNase treatment

- Add 1.5 ul RNase Cocktail Enzyme Mix to each tube

- 37°C 10 minutes incubation
- Move reaction to ice

Cleanup using Ampure XP Beads

- Mix the samples with 17 ul of beads (0.8X, depends on the size of your library, please refer to size selection by Ampure XP beads)
- Mix the beads by flicking
- Incubate 10 minutes at room temperature
- Spin down the tube and place it on the magnet
- Remove the supernatant
- Add 70% freshly prepared 200 ul ethanol to the tube
- Incubate for 30 seconds at room temperature
- Remove the ethanol
- Repeat the washing
- Remove the ethanol completely by spinning down the tube, placing back it on magnet and removing the liquid
- Air-dry the pellet for maximum 1 minute, do not let it dry out completely!
- Resuspend the beads in 16 ul water
- Incubate 5-10 minutes in RT
- Place the beads on magnet
- Transfer the supernatant into a new tube.
- Quantify 1 µl of eluted sample using a Qubit fluorometer

Annealing of Complementary DNA to VNP Oligo

This step is essential to have a double-stranded DNA oligo with an A overhang, which will initiate the ligation to the adapter

Components	Initial Concentration	Final Conc.	Volume
cDNA			15 uL
Tris-Cl pH 7.5	0.1 M	0.01 M	2.25 uL
NaCl	0.5 M	0.05 M	2.25 uL
CompA_DNA	10 uM	0.44 uM	1 uL
Water			2 uL
Total			22.5 uL

- Mix by flicking
- Heat the mixture for 90°C for 1 mins and ramp down to RT at 0.1°C/s (in thermal cycler).
- Mix the following

Barcode Ligation

22.5 uL cDNA-complement mix
2.5 uL Native Barcode (from the EXP-NBD104 kit)

25 uL Blunt/TA Ligase Mix

- Mix by flicking
- Spin down
- Incubate at RT for 10 minutes

Cleanup using Ampure XP Beads

- Mix the samples with 25 ul of beads (0.5X, depends on the size of your library, please refer to size selection by Ampure XP beads)
- Mix the beads by flicking
- Incubate 10 minutes at room temperature
- Spin down the tube and place it on the magnet
- Remove the supernatant
- Add 70% freshly prepared 200 ul ethanol to the tube
- Incubate for 30 seconds at room temperature
- Remove the ethanol
- Repeat the washing
- Remove the ethanol completely by spinning down the tube, placing back it on magnet and removing the liquid
- Air-dry the pellet for maximum 1 minute, do not let it dry out completely!
- Resuspend the beads in 16 ul water (Normally its 26 ul but then in the pooling step it makes to much volume)
- Incubate 5-10 minutes in RT
- Place the beads on magnet
- Transfer the supernatant into a new tube.
- Quantify 1 µl of eluted sample using a Qubit fluorometer
- Pool the barcoded samples at the desired ratio to a final volume of 65 µl in a DNA LoBind 1.5ml Eppendorf tube. Aim for as high a concentration as possible which does not exceed **200 fmoles** total. If the total volume is >65 **µ**l, perform a 2.5x AMPure clean up and elute in 65 **µ**l of nuclease free water.
- Taking the pooled and barcoded DNA, perform adapter ligation as follows, mixing by flicking the tube between each sequential addition.

    - 65 µl 200 fmol pooled barcoded sample
    - 5 µl Adapter Mix II (AMII adapter from the EXP-NBD104 kit)
    - 20 µl NEBNext Quick Ligation Reaction Buffer (5X)
    - 10 µl Quick T4 DNA Ligase

- Mix gently by flicking the tube, and spin down.
- Incubate the reaction for 10 minutes at RT.

Ampure XP Beads Cleanup

- Add 50 ul resuspended AMPure XP beads (0.5X) to the reaction and mix by flicking
- Incubate 10 minutes at room temperature
- Thaw Wash Buffer (WSB) and Elution Buffer (EB) at RT, mix by vortexing, spin down and place on ice. Check if the contents of each tube are clear of any precipitate.
- Spin down the tube and place it on the magnet
- Remove the supernatant

- Add 200 ul WSB to the beads. Close the tube lid, and resuspend the beads by flicking. Return the tube to the magnetic rack, allow beads to pellet and pipette off the supernatant
- Repeat the previous step.
- Remove the WSB completely by spinning down the tube, pipetting out the liquid and placing back it on magnet
- Air-dry the pellet for maximum 1 minute, do not let it dry out completely!
- Resuspend the beads in 13 ul Elution Buffer (EB)
- Incubate 5-10 minutes in RT
- Place the beads on magnet
- Place the elute in a 1.5 ml tube
- Quantify 1 µl of eluted sample using a Qubit fluorometer
- The prepared library is used for loading into the MinION flow cell. Store the library on ice until ready to load.
- Move to the “Prepare the library for loading” step.

Prepare the library for loading

Before starting:

- Thaw the Sequencing Buffer (SQB), Loading Beads (LB), Flush Tether (FLT) and one tube of Flush Buffer (FB)
- Mix the Sequencing Buffer (SQB) and Flush Buffer (FB) tubes by vortexing, spin down and return to ice.
- Spin down the Flush Tether (FLT) tube, mix by pipetting, and return to ice.
- Mix the contents of LB (Loading beads) with a large volume of pipette to make it homogeneous

- In the new tube, prepare the following:
    - 37.5 µl Sequencing Buffer (SQB)
    - 25.5 µl Loading Beads (LB), mixed immediately before use
    - 12 µl DNA library

Priming the flow cell

QC the flowcell:

- Take the flowcell out of the fridge.Connect it to the MinION
- Check Flowcell (QC it to see how many active pores there are)
- Keep it connected until primed.

Prime the flowcell:

- Mix the Sequencing Buffer (SQB) and Flush Buffer (FB) tubes by vortexing, spin down and return to ice.
- Spin down the Flush Tether (FLT) tube, mix by pipetting, and return to ice.
- Remove the bubble from the port
- Open the lid of the nanopore sequencing device and slide the flow cell's priming port cover clockwise so that the priming port is visible.
- Set a P1000 pipette to 200 µl
- Insert the tip into the priming port

- Turn the wheel until the dial shows 220-230 µl, or until you can see a small volume of buffer entering the pipette tip
- Add 30 ul FLT into a tube of FB
- Mix by pipetting up and down
- Load 800 ul of buffer
- Wait for 5 minutes
- Open sample port cover
- Load 200 ul of buffer more into the priming port, observing the bubbles coming out of sample port

Load the library:

- Mix the prepared library gently by pipetting up and down just prior to loading.
- Add 75 µl of sample to the flow cell via the SpotON sample port in a dropwise fashion. Ensure each drop flows into the port before adding the next.
- Gently replace the SpotON sample port cover, making sure the bung enters the SpotON port, close the priming port and replace the MinION lid.

SUPPLEMENTARY TABLES

Supplementary Table 1. Median tail length of the 7 classes of tail types across species and time points

Group	Zebrafish (2HPF)	Zebrafish (4HPF)	Zebrafish (6HPF)	Mouse	Yeast
AllA	40.33	59.82	78.57	93	25.29
IntC	56.98	74.42	79.03	99.28	33
IntG	64,615	83.57	78.53	98	35.51
IntU	55.1	70.02	72,015	90	34
TermC	51,645	54.28	58.75	NA	NA
TermG	49,635	68.1	74.64	82.11	28.54
TermU	42,855	50,455	31.91	46	31.3

Supplementary Table 2. Summary of the Nano3P-seq runs and dRNAseq runs included in this work

Species	RUN ID	Sample Description	Method
SYNTHETIC RNAs	cDNA345234	Curlcakes	Nano3P-seq
YEAST	cDNA231532	Yeast Total RNA	Nano3P-seq
	cDNA365723	Yeast Total RNA	
	cDNA356382	Yeast Total RNA	
MOUSE	cDNA964321	Mouse Nuclear/Mitochondrial	Nano3P-seq
ZEBRAFISH	cDNA8523612	Zebrafish pA selected	Nano3P-seq
	PDBN042841	Zebrafish pA selected	dRNA-seq
	cDNA786327	Zebrafish Ribodepleted Rep1	Nano3P-seq
	cDNA123791	Zebrafish Ribodepleted Rep2	Nano3P-seq
	cDNA897892	Zebrafish Ribodepleted Rep3	Nano3P-seq
HUMAN	cDNA867343	Human pA Selected	Nano3P-seq
SYNTHETIC cDNAs	cDNA925386	cDNA Standards	Nano3P-seq

Spike-in	RNA Template	Replicate	Device	Run Time (hr)
NA	Curlcake 1 (non-polyAed) and Curlcake2 (polyA-ed)	1	Flongle	13
NA	Yeast Total RNA	1	Flongle	20
NA	Yeast Total RNA	2	Flongle	24
NA	Yeast Total RNA	3	Flongle	24
Sequins	Mouse Nuclear/Mitochondrial RNA (rep1)	1	MinION	63
	Mouse Nuclear/Mitochondrial RNA (rep2)	2		
NA	Zebrafish 4 hpf Embryo oligodT-selected RNA	1	Flongle	36
NA	Zebrafish 4 hpf Embryo oligodT-selected RNA	1	PromethION	36
Sequins	Zebrafish 2 hpf Embryo Ribodepleted RNA (rep1)	1	MinION	39
	Zebrafish 4 hpf Embryo Ribodepleted RNA (rep1)			
	Zebrafish 6 hpf Embryo Ribodepleted RNA (rep1)			
Sequins	Zebrafish 2 hpf Embryo Ribodepleted RNA (rep2)	2	MinION	39
	Zebrafish 4 hpf Embryo Ribodepleted RNA (rep2)			
	Zebrafish 6 hpf Embryo Ribodepleted RNA (rep2)			
NA	Zebrafish 2 hpf Embryo Ribodepleted RNA (rep3)	3	MinION	72
NA	Zebrafish 4 hpf Embryo Ribodepleted RNA (rep3)			
NA	Zebrafish 6 hpf Embryo Ribodepleted RNA (rep3)			
NA	HeLa mRNA	1	MinION	72
NA	HeLa mRNA	2		
NA	0 pA standard	1	MinION	22
NA	15 pA standard	1		
NA	30 pA standard	1		
NA	60 pA standard	1		
NA	90 pA standard	1		
NA	120 pA standard	1		
NA	1U + 29 pA standard	1		
NA	3U + 27 pA standard	1		
NA	5U + 25 pA standard	1		
NA	5G + 25 pA standard	1		
NA	5C + 25 pA standard	1		
NA	IntG standard	1		

Barcode	Sequenced reads	Basecalled reads (Guppy 6.0.1)	Demultiplexed reads	Mapped reads (Genome + rRNA)	Mapped reads (Curlcakes/ cDNA standards)	ENA Primary Accession	ENA Sample Accession
NA	60,078	60,078	NA	NA	47,950	PRJEB53494	ERS12199230
NA	257,307	257,307	NA	238,726	NA	PRJEB53494	ERS12199231
NA	269,184	269,184	NA	191,992	NA	PRJEB53494	ERS12199232
NA	123,712	123,712	NA	83,070	NA	PRJEB53494	ERS12199233
1	770,213	770,213	358,351	289,222	NA	PRJEB53494	ERS12199234
2			324,387	151,186	NA	PRJEB53494	ERS12199234
NA	233,101	233,101	NA	207,235	NA	PRJEB53494	ERS12199235
NA	897,768	897,768	NA	363,468	NA	PRJEB53494	ERS12521224
1	1,644,127	1,644,127	340,730	297,460	NA	PRJEB53494	ERS12199236
2			594,200	480,569	NA	PRJEB53494	ERS12199236
3			323,806	302,334	NA	PRJEB53494	ERS12199236
1	1,955,353	1,955,353	628,646	486,072	NA	PRJEB53494	ERS12199237
2			540,195	363,032	NA	PRJEB53494	ERS12199237
3			551,172	333,348	NA	PRJEB53494	ERS12199237
1	587,456	587,456	130,728	109,482	NA	PRJEB53494	ERS12199238
2			181,783	154,695	NA	PRJEB53494	ERS12199238
			189,621	169,293	NA	PRJEB53494	ERS12199238
1	254,740	254,740	121,383	110,186	NA	PRJEB53494	ERS12496709
2			99,102	88,236	NA	PRJEB53494	ERS12496709
1	599,568	599,568	38,271	NA	4,761	PRJEB53494	ERS12199239
2			52,580	NA	8,002	PRJEB53494	ERS12199239
3			54,732	NA	2,100	PRJEB53494	ERS12199239
4			18,911	NA	567	PRJEB53494	ERS12199239
5			15,397	NA	658	PRJEB53494	ERS12199239
6			4,041	NA	57	PRJEB53494	ERS12199239
7			57,810	NA	10,266	PRJEB53494	ERS12199239
8			65,674	NA	11,503	PRJEB53494	ERS12199239
9			36,937	NA	5,879	PRJEB53494	ERS12199239
10			53,576	NA	9,213	PRJEB53494	ERS12199239
11			55,884	NA	12,584	PRJEB53494	ERS12199239
12			58,789	NA	8,999	PRJEB53494	ERS12199239

Supplementary Table 3 Correlation values between per-gene polyA tail length estimations between different methods and Nano3P-seq

Pearson

Human (HeLa)

	FLAM-seq	PAL-seq	TAIL-seq	Nano3P-seq
FLAM-seq	-	0.31	0.10	0.47
PAL-seq	0.31	-	0.19	0.47
TAIL-seq	0.10	0.19	-	0.19
Nano3P-seq	0.47	0.47	0.19	-

Yeast

Methods	PAT-seq	PAL-seq	Nano3P-seq
PAT-seq	-	0.42	0.43
PAL-seq	0.42	-	0.43
Nano3P-seq	0.43	0.43	-

Zebrafish

Timepoints	Nano3P-seq vs PAL-seq
2 hpf	0.82
4 hpf	0.85
6 hpf	0.71

Spearman

Human (HeLa)

	FLAM-seq	PAL-seq	TAIL-seq	Nano3P-seq
FLAM-seq	-	0.31	0.10	0.43
PAL-seq	0.31	-	0.18	0.44
TAIL-seq	0.10	0.18	-	0.17
Nano3P-seq	0.43	0.44	0.17	-

Yeast

Methods	PAT-seq	PAL-seq	Nano3P-seq
PAT-seq	-	0.43	0.44
PAL-seq	0.43	-	0.44
Nano3P-seq	0.44	0.44	-

Zebrafish

Timepoints	Nano3P-seq vs PAL-seq
2 hpf	0.82
4 hpf	0.69
6 hpf	0.86

SUPPLEMENTARY NOTES

Supplementary Note S1.

Tail composition analyses in cDNA standards (**Figure 5c** and **Extended Data Figure 5a**) revealed slight inaccuracies in the predictions, mainly in cDNA standards containing 5U, 5C or 5G at their 3'ends. We believe that this phenomenon is caused by the homopolymeric nature of these sequences. Indeed, previous reports have shown that increased base-calling errors (in the form of insertions and/or deletions) in nanopore sequencing datasets frequently occur at homopolymeric positions consisting of 5 or more identical consecutive bases(Delahaye and Nicolas 2021; Huang, Liu, and Shih 2021). These inaccuracies are unlikely to be seen in biological systems, since Nano3P-seq (and previous literature (Legnini et al. 2019; Chang et al. 2014)) in *in vivo* datasets shows that the majority of homopolymeric non-A terminal ends are shorter than 5 bases.

Supplementary Note S2.

Although the chromatogram in Extended Data Figure 2j only shows 4 A nucleosides based on the "consensus prediction", there are additional A bases that can also be seen in subsequent positions, which have smaller peaks than the G peaks, with decreasing frequency. This phenomenon is the result of sequencing a heterogeneous population of polyA tail lengths, where G/I tails are incorporated into RNA populations with slight differences in their polyA tail lengths.

Supplementary Note References

Chang, Hyeshik, Jaechul Lim, Minju Ha, and V. Narry Kim. 2014. "TAIL-Seq: Genome-Wide Determination of poly(A) Tail Length and 3' End Modifications." *Molecular Cell* 53 (6): 1044–52.

Delahaye, Clara, and Jacques Nicolas. 2021. "Sequencing DNA with Nanopores: Troubles and Biases." *PloS One* 16 (10): e0257521.

Harrison, Paul F., David R. Powell, Jennifer L. Clancy, Thomas Preiss, Peter R. Boag, Ana Traven, Torsten Seemann, and Traude H. Beilharz. 2015. "PAT-Seq: A Method to Study the Integration of 3'-UTR Dynamics with Gene Expression in the Eukaryotic Transcriptome." *RNA* 21 (8): 1502–10.

Huang, Yao-Ting, Po-Yu Liu, and Pei-Wen Shih. 2021. "Homopolish: A Method for the Removal of Systematic Errors in Nanopore Sequencing by Homologous Polishing." *Genome Biology* 22 (1): 95.

Legnini, Ivano, Jonathan Alles, Nikos Karaiskos, Salah Ayoub, and Nikolaus Rajewsky. 2019. "FLAM-Seq: Full-Length mRNA Sequencing Reveals Principles of poly(A) Tail Length Control." *Nature Methods* 16 (9): 879–86.

Lim, Jaechul, Minju Ha, Hyeshik Chang, S. Chul Kwon, Dhirendra K. Simanshu, Dinshaw J. Patel, and V. Narry Kim. 2014. "Uridylation by TUT4 and TUT7 Marks mRNA for Degradation." *Cell* 159 (6): 1365–76.

Subtelny, Alexander O., Stephen W. Eichhorn, Grace R. Chen, Hazel Sive, and David P. Bartel. 2014. "Poly(A)-Tail Profiling Reveals an Embryonic Switch in Translational Control." *Nature* 508 (7494): 66–71.
