# Supplementary material for: Nano3P-seq: transcriptome-wide analysis of gene expression and tail dynamics using end-capture nanopore cDNA sequencing
Source: Nat Methods. 2022 Dec 19;20(1):75–85. doi: 10.1038/s41592-022-01714-w (PMC9834059; doi:10.1038/s41592-022-01714-w)
Supplement: Supplementary file 2 — Reporting Summary [file 41592_2022_1714_MOESM2_ESM.pdf]

nature portfolio

Corresponding author(s): Eva Maria Novoa Pardo

Last updated by author(s): Oct 6, 2022

Reporting Summary

Nature Portfolio wishes to improve the reproducibility of the work that we publish. This form provides structure for consistency and transparency in reporting. For further information on Nature Portfolio policies, see our Editorial Policies and the Editorial Policy Checklist.

Statistics

For all statistical analyses, confirm that the following items are present in the figure legend, table legend, main text, or Methods section.

n/a	Confirmed	
☐	☒	The exact sample size (*n*) for each experimental group/condition, given as a discrete number and unit of measurement
☐	☒	A statement on whether measurements were taken from distinct samples or whether the same sample was measured repeatedly
☐	☒	The statistical test(s) used AND whether they are one- or two-sided<br>*Only common tests should be described solely by name; describe more complex techniques in the Methods section.*
☐	☒	A description of all covariates tested
☐	☒	A description of any assumptions or corrections, such as tests of normality and adjustment for multiple comparisons
☐	☒	A full description of the statistical parameters including central tendency (e.g. means) or other basic estimates (e.g. regression coefficient) AND variation (e.g. standard deviation) or associated estimates of uncertainty (e.g. confidence intervals)
☐	☒	For null hypothesis testing, the test statistic (e.g. *F*, *t*, *r*) with confidence intervals, effect sizes, degrees of freedom and *P* value noted<br>*Give P values as exact values whenever suitable.*
☒	☐	For Bayesian analysis, information on the choice of priors and Markov chain Monte Carlo settings
☒	☐	For hierarchical and complex designs, identification of the appropriate level for tests and full reporting of outcomes
☐	☒	Estimates of effect sizes (e.g. Cohen's *d*, Pearson's *r*), indicating how they were calculated

Our web collection on statistics for biologists contains articles on many of the points above.

Software and code

Policy information about availability of computer code

Data collection	Nanopore data was acquired using Minknow software v.3.5.5 .
Data analysis	Nanopore data was basecalled using Guppy version 4.0. TailfindR tool (Nano3P-seq mode) was used for tail length estimation. Minimap2 and BWA tools were used for mapping the basecalled nanopore reads. Code used in this study is made available in the following link : https://github.com/novoalab/Nano3P_Seq. TailfindR nano3pseq mode can be accessed using this link : https://github.com/adnaniazi/tailfindr/tree/nano3p-seq.

For manuscripts utilizing custom algorithms or software that are central to the research but not yet described in published literature, software must be made available to editors and reviewers. We strongly encourage code deposition in a community repository (e.g. GitHub). See the Nature Portfolio guidelines for submitting code & software for further information.

Data

Policy information about availability of data

All manuscripts must include a data availability statement. This statement should provide the following information, where applicable:

- Accession codes, unique identifiers, or web links for publicly available datasets
- A description of any restrictions on data availability
- For clinical datasets or third party data, please ensure that the statement adheres to our policy

Base-called FAST5 reads from Nano3P-seq and dRNA-seq libraries are publicly available in ENA, under accession code PRJEB53494. PAL-Seq polyA tail length

estimates used in this work were obtained from GEO with the accession code GSE52809. PAT-seq polyA tail length estimates used in this work were obtained from GEO with the accession code GSE53461. FLAM-seq polyA tail length estimates used in this work were obtained from GEO with the accession code GSE126465 TAIL-seq polyA tail length estimates used in this work were obtained from GEO with the accession code GSE51299.

Human research participants

Policy information about studies involving human research participants and Sex and Gender in Research.

Reporting on sex and gender	*Use the terms sex (biological attribute) and gender (shaped by social and cultural circumstances) carefully in order to avoid confusing both terms. Indicate if findings apply to only one sex or gender; describe whether sex and gender were considered in study design whether sex and/or gender was determined based on self-reporting or assigned and methods used. Provide in the source data disaggregated sex and gender data where this information has been collected, and consent has been obtained for sharing of individual-level data; provide overall numbers in this Reporting Summary. Please state if this information has not been collected. Report sex- and gender-based analyses where performed, justify reasons for lack of sex- and gender-based analysis.*
Population characteristics	*Describe the covariate-relevant population characteristics of the human research participants (e.g. age, genotypic information, past and current diagnosis and treatment categories). If you filled out the behavioural & social sciences study design questions and have nothing to add here, write "See above."*
Recruitment	*Describe how participants were recruited. Outline any potential self-selection bias or other biases that may be present and how these are likely to impact results.*
Ethics oversight	*Identify the organization(s) that approved the study protocol.*

Note that full information on the approval of the study protocol must also be provided in the manuscript.

Field-specific reporting

Please select the one below that is the best fit for your research. If you are not sure, read the appropriate sections before making your selection.

☒ Life sciences ☐ Behavioural & social sciences ☐ Ecological, evolutionary & environmental sciences

For a reference copy of the document with all sections, see nature.com/documents/nr-reporting-summary-flat.pdf

Life sciences study design

All studies must disclose on these points even when the disclosure is negative.

Sample size	Sample size was chosen as n=3 for most experiments, allowing to perform statistical tests to assess significance in the observed differences between groups. Sample size in this case corresponds to biological replicates
Data exclusions	No data was excluded from our analyses.
Replication	In-vitro constructs (RNA and cDNA) were sequenced once. S. cerevisiae data has three biological replicates. Mus musculus data has two biological replicates. Danio rerio has three replicates for three time-points. HeLa (Human cell line) has two biological replicates.
Randomization	Randomization is not relevant to our study.
Blinding	Blinding was not relevant to our study.

Reporting for specific materials, systems and methods

We require information from authors about some types of materials, experimental systems and methods used in many studies. Here, indicate whether each material, system or method listed is relevant to your study. If you are not sure if a list item applies to your research, read the appropriate section before selecting a response.

Materials & experimental systems

n/a	Involved in the study	
☒	☐	Antibodies
☐	☒	Eukaryotic cell lines
☒	☐	Palaeontology and archaeology
☐	☒	Animals and other organisms
☒	☐	Clinical data
☒	☐	Dual use research of concern

Methods

n/a	Involved in the study	
☒	☐	ChIP-seq
☒	☐	Flow cytometry
☒	☐	MRI-based neuroimaging

Eukaryotic cell lines

Policy information about cell lines and Sex and Gender in Research

Cell line source(s)	HeLa-CCL-2 was purchased from ATCC.
Authentication	None of the cell lines were authenticated.
Mycoplasma contamination	Cell lines were tested for mycoplasma contamination.
Commonly misidentified lines (See ICLAC register)	No commonly misidentified samples were used in this study.

Animals and other research organisms

Policy information about studies involving animals; ARRIVE guidelines recommended for reporting animal research, and Sex and Gender in Research

Laboratory animals	Mus musculus and Danio rerio were used in this study. Experiments were performed with male mice aged between 8 and 10 weeks. All mice were euthanized using CO2 and tissues were snap frozen in liquid nitrogen. Animals were kept on a 12:12h light:dark cycle and provided with water and food ad libitum. Wild-type zebrafish (Danio rerio) embryos were obtained through natural mating of the TU-AB strain of mixed ages (5–18 months). Mating pairs were randomly chosen from a pool of 60 males and 60 females allocated for each day of the month. Embryos and adult fish were maintained at 28°C.
Wild animals	No wild animals were used in this study.
Reporting on sex	Male mouse were used in this study.
Field-collected samples	No field collected samples were used in this study.
Ethics oversight	Fish lines were maintained according to the International Association for Assessment and Accreditation of Laboratory Animal Care research guidelines, and protocols were approved by the Yale University Institutional Animal Care and Use Committee (IACUC). Mice maintenance was approved by the Garvan/St Vincent's Hospital Animal Ethics Committee, in accordance with the guidelines of the Australian Code of Practice for the Care and Use of Animals for Scientific Purposes (Project No. 16/14 and 16/26).

Note that full information on the approval of the study protocol must also be provided in the manuscript.
