# Supplementary figures and images for: Nano3P-seq: transcriptome-wide analysis of gene expression and tail dynamics using end-capture nanopore cDNA sequencing
Source: Nat Methods. 2022 Dec 19;20(1):75–85. doi: 10.1038/s41592-022-01714-w (PMC9834059; doi:10.1038/s41592-022-01714-w)

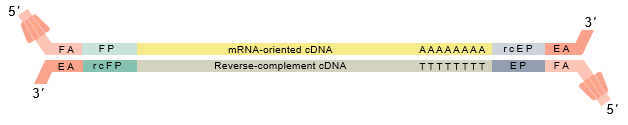

Supplement: Supplementary file 4 — Code corresponding to TailfindR Nano3P-seq branch found in GitHub repository (attached as a separate zip file). This version incorporates the custom adapter sequences that are used in Nano3P-seq library preparation protocols. [file 41592_2022_1714_MOESM4_ESM.zip › tailfindr-nano3p-seq/man/figures/cdna_construct.png]

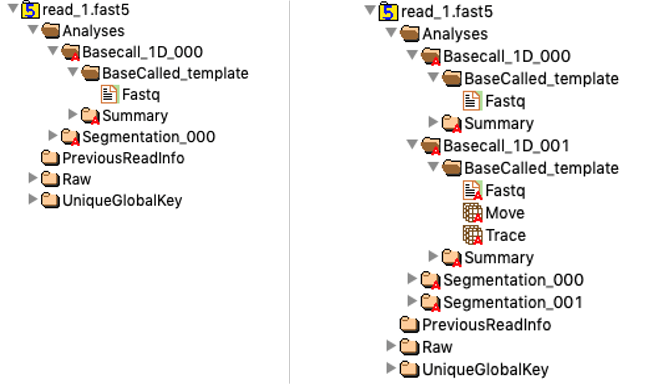

Supplement: Supplementary file 4 — Code corresponding to TailfindR Nano3P-seq branch found in GitHub repository (attached as a separate zip file). This version incorporates the custom adapter sequences that are used in Nano3P-seq library preparation protocols. [file 41592_2022_1714_MOESM4_ESM.zip › tailfindr-nano3p-seq/man/figures/minkow_live_basecalling.png]

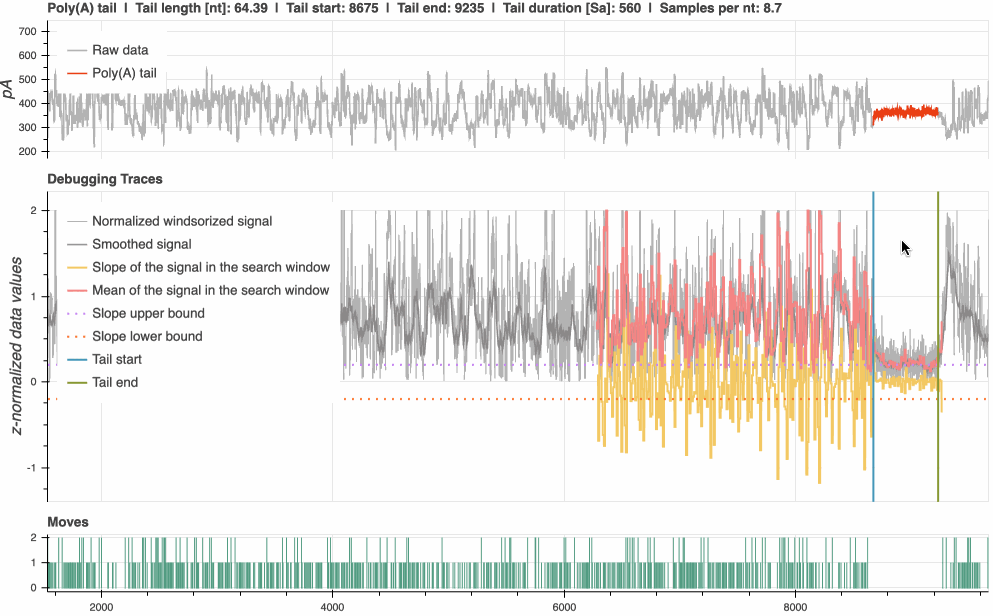

Supplement: Supplementary file 4 — Code corresponding to TailfindR Nano3P-seq branch found in GitHub repository (attached as a separate zip file). This version incorporates the custom adapter sequences that are used in Nano3P-seq library preparation protocols. [file 41592_2022_1714_MOESM4_ESM.zip › tailfindr-nano3p-seq/man/figures/poly_a_with_debug.gif]

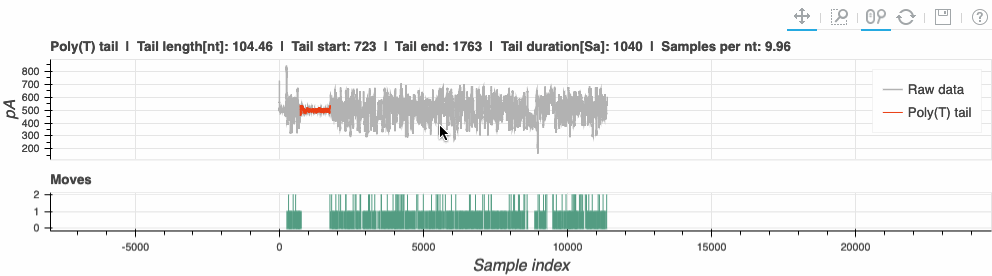

Supplement: Supplementary file 4 — Code corresponding to TailfindR Nano3P-seq branch found in GitHub repository (attached as a separate zip file). This version incorporates the custom adapter sequences that are used in Nano3P-seq library preparation protocols. [file 41592_2022_1714_MOESM4_ESM.zip › tailfindr-nano3p-seq/man/figures/poly_t_without_debug.gif]

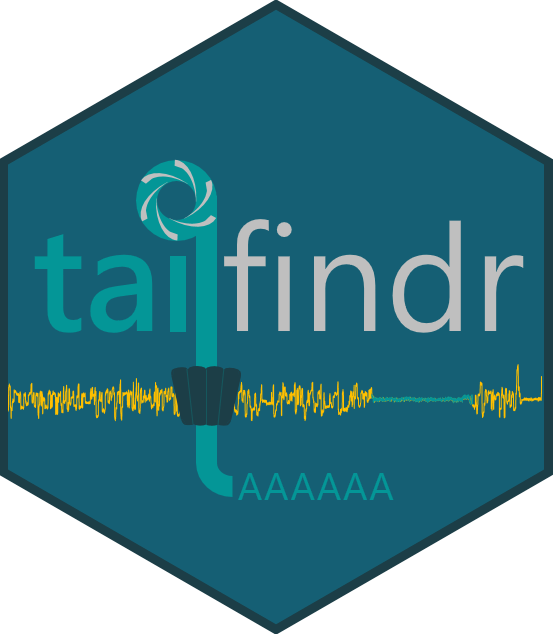

Supplement: Supplementary file 4 — Code corresponding to TailfindR Nano3P-seq branch found in GitHub repository (attached as a separate zip file). This version incorporates the custom adapter sequences that are used in Nano3P-seq library preparation protocols. [file 41592_2022_1714_MOESM4_ESM.zip › tailfindr-nano3p-seq/man/figures/tailfindr-logo.png]
